# Supplementary material for: Stem-loop-induced ribosome queuing in the uORF2/ATF4 overlap fine-tunes stress-induced human ATF4 translational control
Source: Cell Rep. Author manuscript; Available in PMC 2024 Apr 30. (PMC11058473; doi:10.1016/j.celrep.2024.113976)
Supplement: SupplementaryMaterial [file NIHMS1986405-supplement-SupplementaryMaterial.zip › 1-s2.0-S2211124724003048-mmc1.pdf]

Cell Reports, Volume *43*

Supplemental information

Stem-loop-induced ribosome queuing in the uORF2/*ATF4* overlap fine-tunes stress-induced human ATF4 translational control

Anna M. Smirnova, Vladislava Hronová, Mahabub Pasha Mohammad, Anna Herrmannová, Stanislava Gunišová, Denisa Petráčková, Petr Halada, Štěpán Coufal, Michał Świrski, Justin Rendleman, Kristína Jendruchová, Maria Hatzoglou, Petra Beznosková, Christine Vogel, and Leoš Shivaya Valášek

SUPPLEMENTAL INFORMATION

SUPPLEMENTARY FIGURES AND FIGURE LEGENDS

A

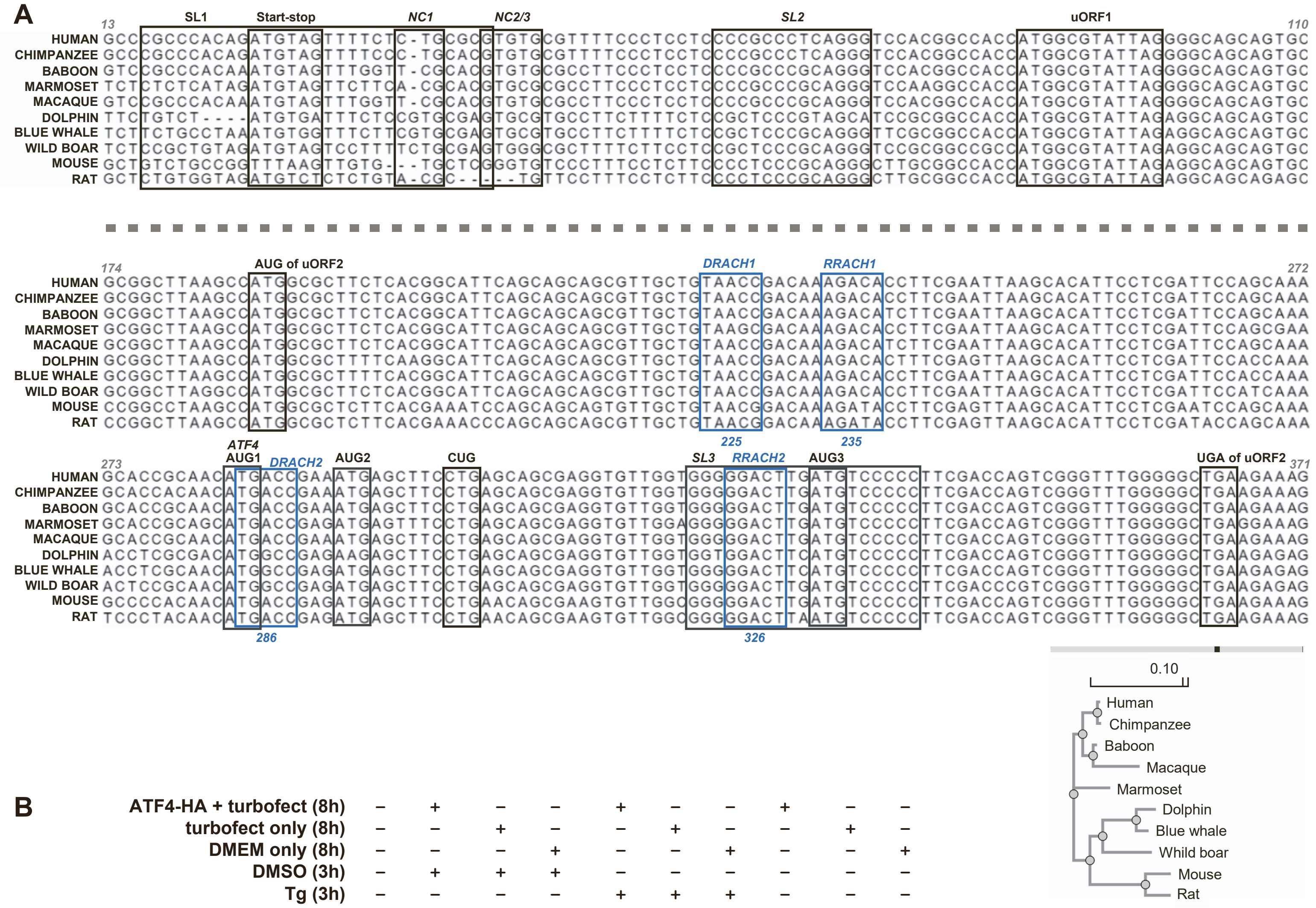

B

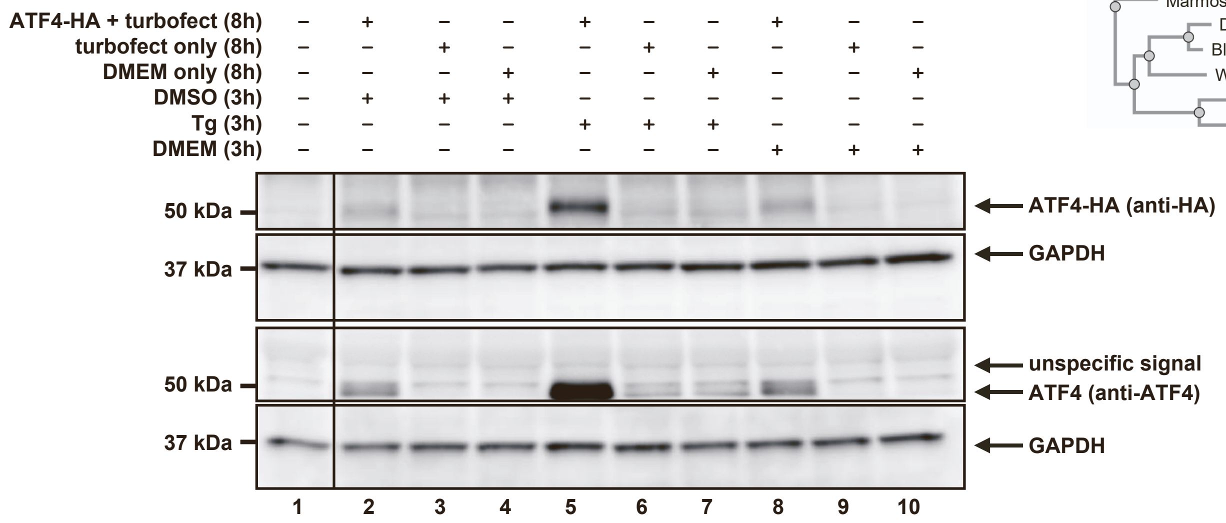

C

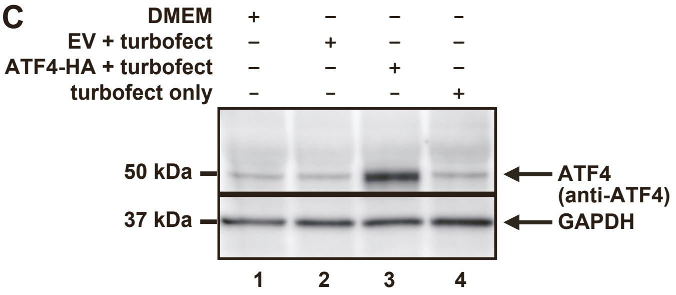

D

fold induction of ATF4 expression in Tg stress over non-stress conditions

JESS Protein Simple		Western Blot	
anti-HA	anti-ATF4	anti-HA	anti-ATF4
5.21 ± 0.5	5.52 ± 0.6	3.90 ± 1.3	3.40 ± 0.2

Figure S1. The SL3 and CUG are conserved in evolution (related to Figures 1 and 2 and text in Methods Details)**.**

(A) Multiple Sequence Alignment of 10 mammalian species using MAFFT (Multiple Alignment using Fast Fourier Transform) is shown for the 5′ UTR of ATF4 mRNA beginning 12 nucleotides upstream of the AUG of Start-stop and ending six nucleotides past the stop codon of uORF2. The ATF4‘s mRNA-specific features under study and their conservation comparisons are outlined in bold boxes. Genomic alignments of mammalian species were compared using Job Dispatcher EMBL-EBI website MAFFT tool and Madeira et al.[1]. The Phylogram based on Phylogenetic Tree scores reflects sequence differences between the *ATF4* gene among given species.

(B) Our ATF4-HA reporter system faithfully recapitulates the endogenous *ATF4* regulation as demonstrated by traditional western blot analysis. See STAR Methods for further details. Results are representative of three independent experiments.

(C) Transfection of an empty vector (EV) did not increase ATF4 expression under non-stress conditions as demonstrated by traditional western blot analysis. See text for further details. Results are representative of three independent experiments.

(D) Fold induction of ATF4 expression in Tg stress *versus* non-stress conditions is comparable over different methods and antibodies. All quantifications were done from a minimum of three independent experiments (JESS anti-HA n=17; JESS anti-ATF4 n=3; WB anti-HA n=3; WB anti-ATF4 n=4). For western blots, only exposures with non-saturated signals were used for quantifications by Quantity One software.

A

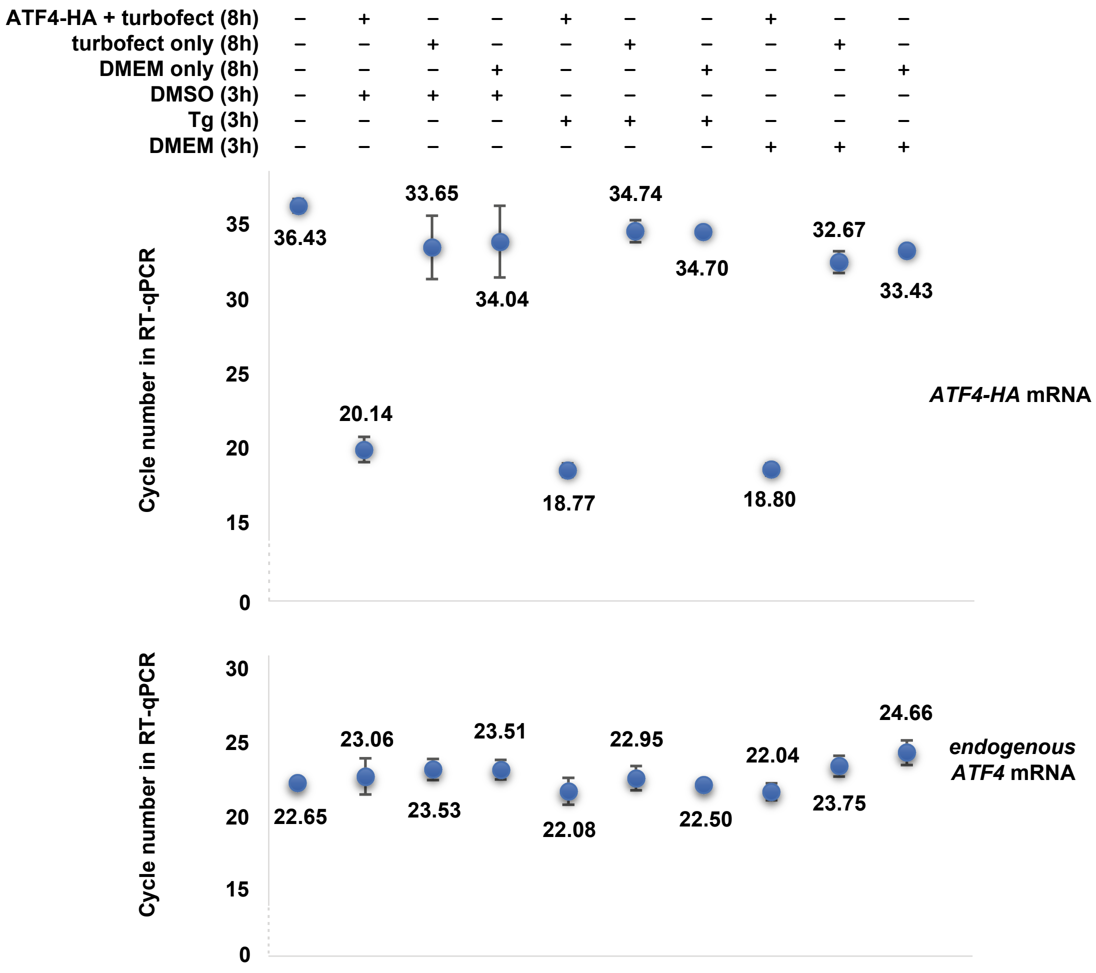
ATF4-HA + turbofect (8h)
turbofect only (8h)
DMEM only (8h)
DMSO (3h)
Tg (3h)
DMEM (3h)
Cycle number in RT-qPCR
36.43
20.14
33.65
34.04
18.77
34.74
34.70
18.80
32.67
33.43
ATF4-HA mRNA
22.65
23.06
23.53
23.51
22.08
22.95
22.50
22.04
23.75
24.66
endogenous
ATF4 mRNA

B

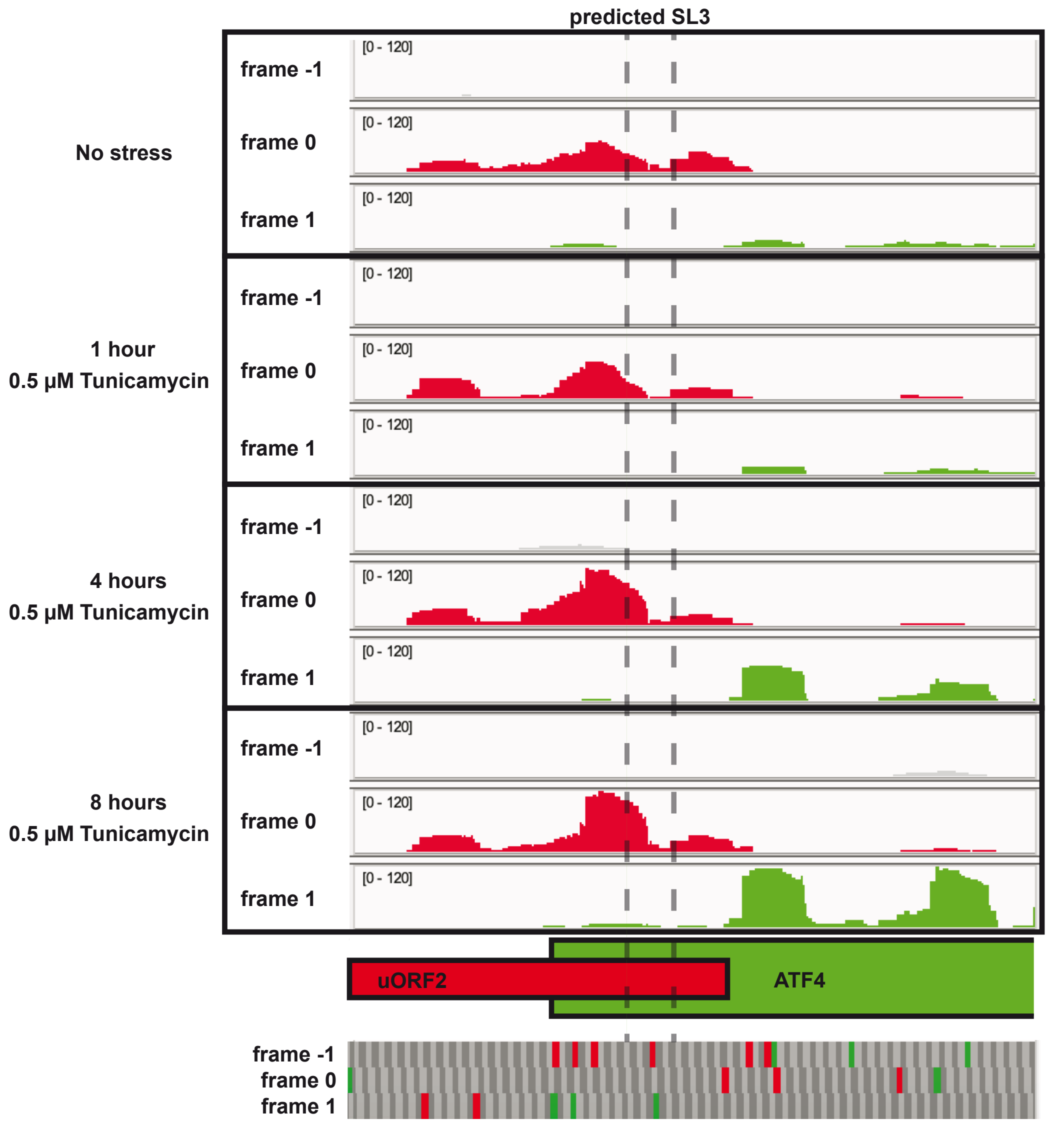
predicted SL3
No stress
1 hour
0.5 μM Tunicamycin
4 hours
0.5 μM Tunicamycin
8 hours
0.5 μM Tunicamycin
frame -1
frame 0
frame 1
[0 - 120]
uORF2
ATF4

Figure S2. Our ATF4-HA reporter system faithfully recapitulates the endogenous *ATF4* regulation; does uORF2 to *ATF4* frameshifting occurs during acute stress (related to Figures 1 and 2 and text in Methods Details)**?**

(A) mRNA levels of both *ATF4-HA* and endogenous *ATF4* (measured separately using highly specific reverse primers matching the *ATF4* stop codon region, by which these two alleles differ due to the HA tag sequence) remain virtually unchanged under non-stress *vs.* stress conditions. See text for further details.

(B) Ribosome footprints from HeLa cells undergoing acute ER stress[2] were mapped to the *ATF4* exon 2 based on the reading frame engaged. Reading frame assignment was determined by the +12 position in reads 28-30 bp long, corresponding to the P-site of ribosomes. ER stress was induced by 0.5 μM Tunicamycin and cells were harvested at 0, 1, 4, and 8 hours post-treatment, as described in Rendleman et al.[2]. Predicted stem-loop 3 (SL3) start and end positions are indicated by the dashed line. Below, canonical start and stop codons within frames -1, 0, and 1 are indicated in green and red, respectively.

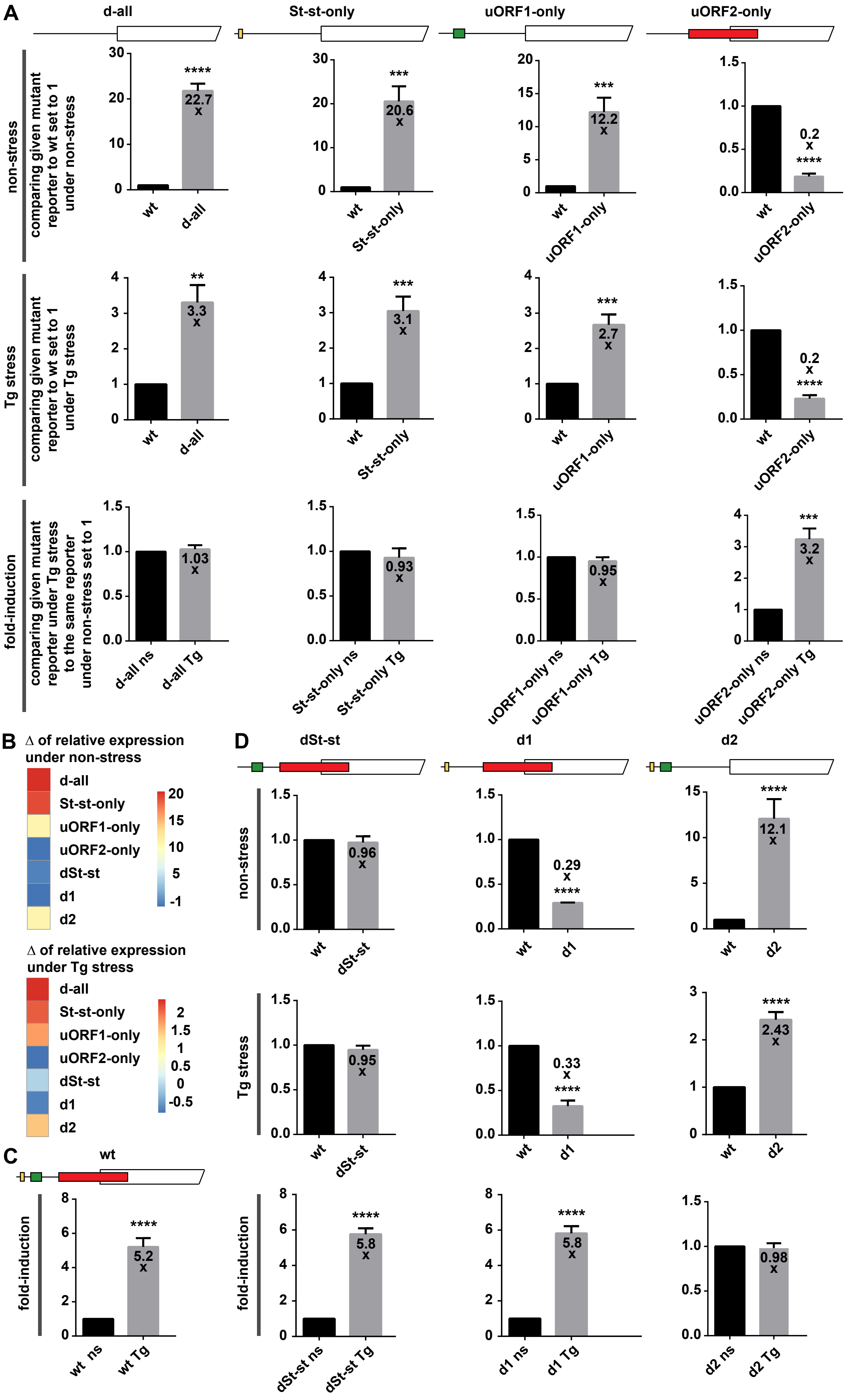
A
d-all
St-st-only
uORF1-only
uORF2-only
non-stress
comparing given mutant reporter to wt set to 1 under non-stress
Tg stress
comparing given mutant reporter to wt set to 1 under Tg stress
fold-induction
comparing given mutant reporter under Tg stress to the same reporter under non-stress set to 1
22.7 x
20.6 x
12.2 x
0.2 x
3.3 x
3.1 x
2.7 x
0.2 x
1.03 x
0.93 x
0.95 x
3.2 x
wt
d-all
St-st-only
uORF1-only
uORF2-only
d-all ns
d-all Tg
St-st-only ns
St-st-only Tg
uORF1-only ns
uORF1-only Tg
uORF2-only ns
uORF2-only Tg
B
∆ of relative expression under non-stress
d-all
St-st-only
uORF1-only
uORF2-only
dSt-st
d1
d2
∆ of relative expression under Tg stress
C
wt
fold-induction
5.2 x
wt ns
wt Tg
D
dSt-st
d1
d2
non-stress
Tg stress
fold-induction
0.96 x
0.29 x
12.1 x
0.95 x
0.33 x
2.43 x
5.8 x
5.8 x
0.98 x
dSt-st ns
dSt-st Tg
d1 ns
d1 Tg
d2 ns
d2 Tg

Figure S3. Revisiting the delayed translation reinitiation mechanism of *ATF4* translational control – uORF2 shows an inducible nature under Tg stress (related to Figure 1 and text in Methods Details)**.**

(A) Schematics at the top of the corresponding panels indicate the *ATF4* mutant constructs subjected to JESS analyses. Relative ATF4-HA protein expression levels were plotted as ratios of values obtained with mutants *versus* wt set to 1 under "non-stress" and "Tg stress" conditions; "fold-induction" plots depict ratios of Tg stress *versus* non-stress values obtained with mutant constructs. Statistical analyses were carried out as in Figure 1E (n≥3).

(B) Heatmaps, created in R (ver. 4.2.2) using the 'pheatmap' package (ver. 1.0.12)[3], show the differences (Δ) in the relative ATF4-HA protein expression of individual mutant *versus* wt constructs that were obtained under non- stress (top panel) and Tg stress conditions (bottom panel). The maximal expression levels are shown in red (for the control d-all construct), whereas the minimal levels are depicted in blue (for the uORF2-only construct).

(C) Same as Figure 1E for better comparison.

(D) Same as panel A with different constructs, depicted at the top, under study.

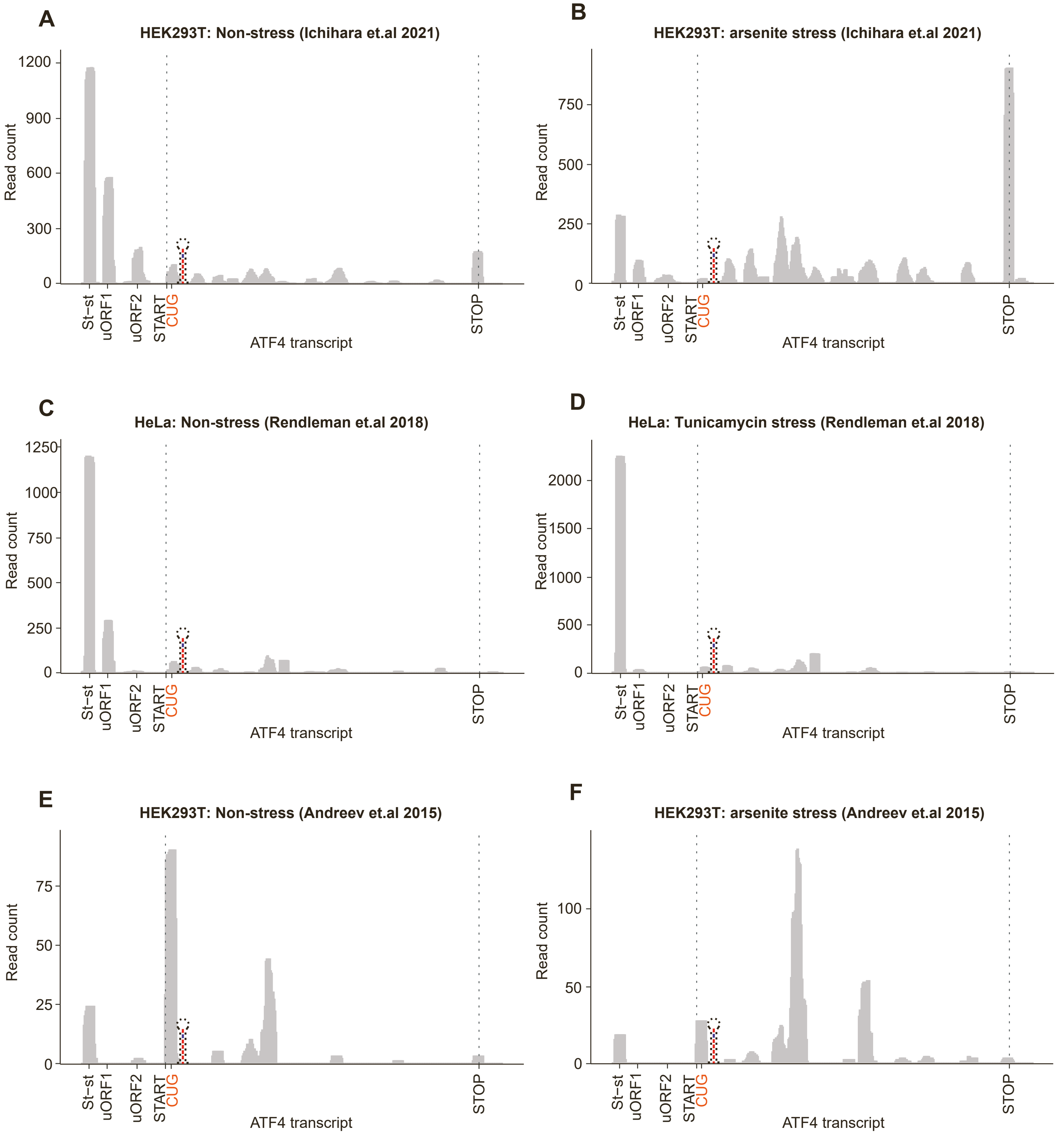
A
HEK293T: Non-stress (Ichihara et.al 2021)
Read count
1200
900
600
300
0
St−st
uORF1
uORF2
START
CUG
STOP
ATF4 transcript
B
HEK293T: arsenite stress (Ichihara et.al 2021)
Read count
750
500
250
0
St−st
uORF1
uORF2
START
CUG
STOP
ATF4 transcript
C
HeLa: Non-stress (Rendleman et.al 2018)
Read count
1250
1000
750
500
250
0
St−st
uORF1
uORF2
START
CUG
STOP
ATF4 transcript
D
HeLa: Tunicamycin stress (Rendleman et.al 2018)
Read count
2000
1500
1000
500
0
St−st
uORF1
uORF2
START
CUG
STOP
ATF4 transcript
E
HEK293T: Non-stress (Andreev et.al 2015)
Read count
75
50
25
0
St−st
uORF1
uORF2
START
CUG
STOP
ATF4 transcript
F
HEK293T: arsenite stress (Andreev et.al 2015)
Read count
100
50
0
St−st
uORF1
uORF2
START
CUG
STOP
ATF4 transcript

Figure S4. SL3 stalls the ribosome in the uORF2/*ATF4* overlap region (related to Figures 2 – 4)**.** The profiles show ribosome foot-print coverage on the *ATF4* mRNA generated by RiboWaltz[4] package using the datasets obtained from three different studies examining HEK293T cells treated with DMSO (A and E) or Sodium arsenite (B and F) or HeLa cells treated with tunicamycin for 0 (C) or 4 h (D)[2,5,6]. The *ATF4* transcript with its *cis*-acting features indicated is plotted on the X-axis. Raw read count is plotted on the Y-axis. The dotted lines indicate the 1st nucleotide of canonical start and stop codons of the *ATF4* ORF; the near cognate "CUG" codon is highlighted in red. The schematic stem-loop indicates the location of the predicted RNA secondary structure designed SL3.

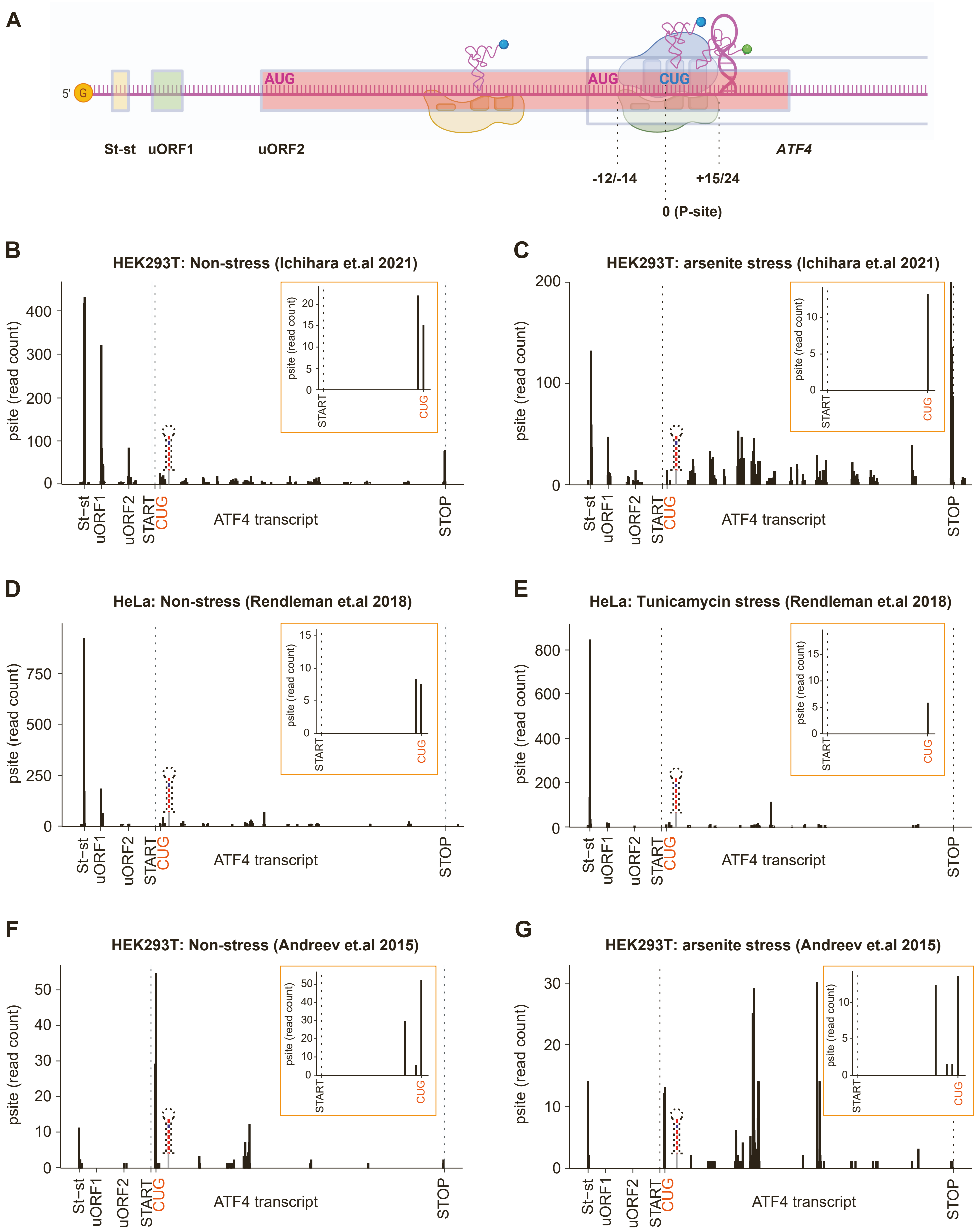
A
5'
G
AUG
AUG
CUG
St-st
uORF1
uORF2
ATF4
-12/-14
+15/24
0 (P-site)
B
HEK293T: Non-stress (Ichihara et.al 2021)
psite (read count)
St-st
uORF1
uORF2
START
CUG
ATF4 transcript
STOP
C
HEK293T: arsenite stress (Ichihara et.al 2021)
D
HeLa: Non-stress (Rendleman et.al 2018)
E
HeLa: Tunicamycin stress (Rendleman et.al 2018)
F
HEK293T: Non-stress (Andreev et.al 2015)
G
HEK293T: arsenite stress (Andreev et.al 2015)

Figure S5. SL3 may promote placement of the near-cognate "CUG" in the ribosomal P-site (related to Figures 2 – 4).

(A) Schematic illustrating that the ribosome paused by SL3 on *ATF4* mRNA is in an ideal distance to position the near-cognate CUG in its P site to initiate translation.

(B – G) The profiles display the P-site occupancy of Ribosome Protected Fragments (RPFs) on the *ATF4* mRNA generated by RiboWaltz[4] package using the datasets obtained from three different studies examining HEK293T cells treated with DMSO (B and F) or Sodium arsenite (C and G) or HeLa cells treated with tunicamycin for 0 (D) or 4 h (E)[2,5,6]. The corresponding zoomed-in views of the P-site occupancy at the near-cognate "CUG" codon are shown in orangish boxes. The *ATF4* transcript with its *cis*-acting features indicated is plotted on the X-axis. Raw read count is plotted on the Y-axis. The dotted lines indicate the 1st nucleotide of canonical start and stop codons of the *ATF4* ORF; the near cognate "CUG" codon is highlighted in red. The schematic of the stem-loop indicates the location of the predicted RNA secondary structure designated as SL3.

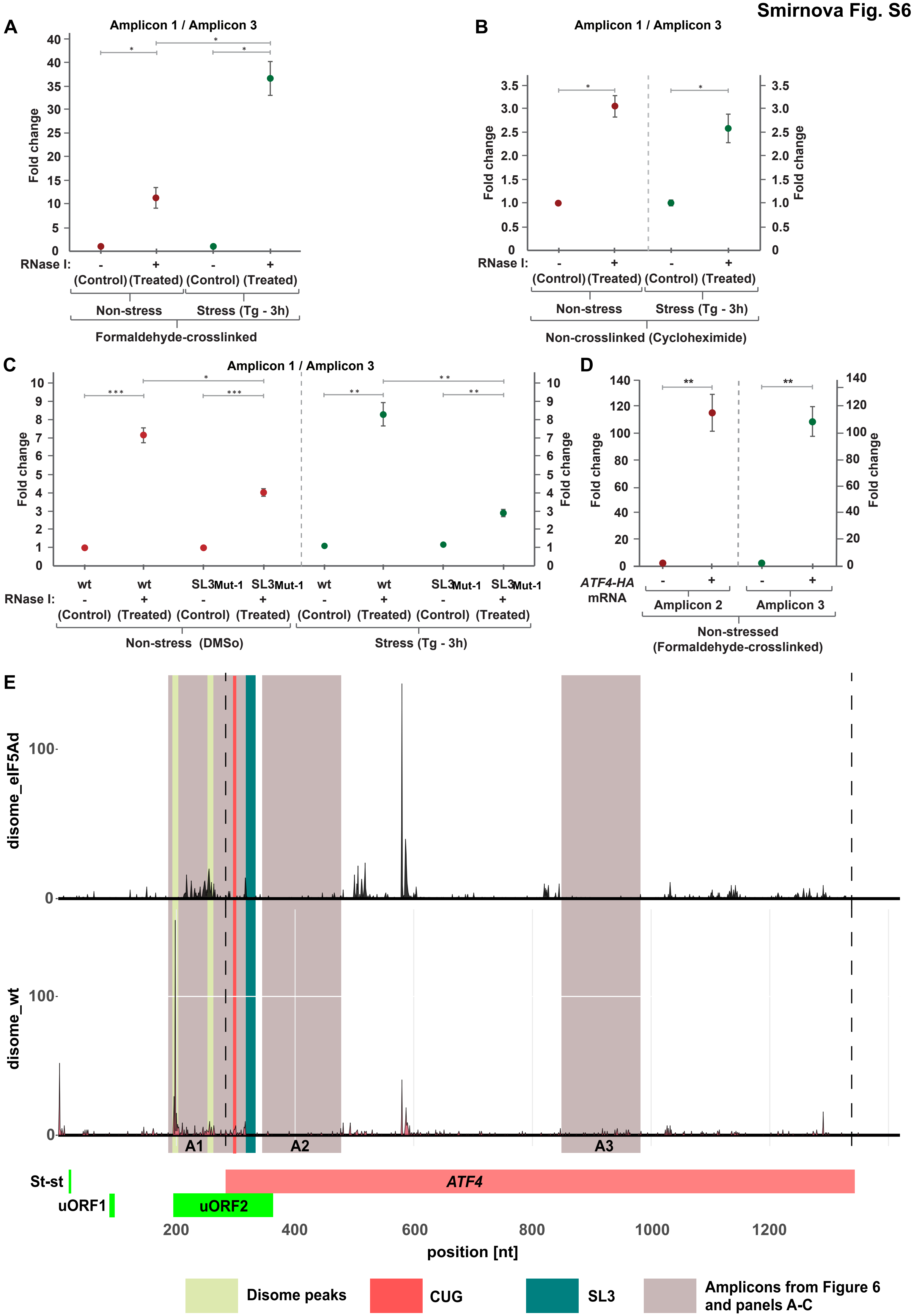

A
Amplicon 1 / Amplicon 3
Fold change
RNase I:
(Control) (Treated) (Control) (Treated)
Non-stress
Stress (Tg - 3h)
Formaldehyde-crosslinked
B
Amplicon 1 / Amplicon 3
Fold change
RNase I:
(Control) (Treated) (Control) (Treated)
Non-stress
Stress (Tg - 3h)
Non-crosslinked (Cycloheximide)
C
Amplicon 1 / Amplicon 3
Fold change
wt
SL3Mut-1
RNase I:
(Control) (Treated) (Control) (Treated) (Control) (Treated) (Control) (Treated)
Non-stress (DMSo)
Stress (Tg - 3h)
D
Fold change
ATF4-HA mRNA
Amplicon 2
Amplicon 3
Non-stressed (Formaldehyde-crosslinked)
E
disome_eIF5Ad
disome_wt
A1
A2
A3
St-st
uORF1
uORF2
ATF4
position [nt]
Disome peaks
CUG
SL3
Amplicons from Figure 6 and panels A-C

Figure S6. Further evidence supporting ribosome queuing hypothesis (related to Figures 5 and 6)**.**
(A) HEK293T cells were cross-linked with formaldehyde (HCHO) and then subjected to the ribosome-protection assay as described in panel B of Figure 7. qPCR product levels of the recovered putative queuing region (amplicon A1) are normalized to the region in the middle of the *ATF4* CDS (amplicon A3), as well as to the internal RNA isolation control (SPIKE) with non-stress values set to 1 (*p<0.01). Results are representative of three independent experiments.
(B) HEK293T cells were treated with cycloheximide (non-crosslinking agent) then subjected to ribosome-protection assay as described in panel C of Figure 7. Results from three independent experiments were analyzed as described in panel B of Figure 7 with non-stress values set to 1 (*p<0.01).
(C) HEK293T cells were transiently transfected with plasmids carrying either wt or SL3-mutated (in $SL3^{Mut\text{-}1}$) *ATF4* reporters and treated as described in panel B of Figure 7. Results three independent experiments were analyzed as described in panel B of Figure 7 with the wt values set to 1 (*p<0.01, **p<0.001, ***p<0.0001).
(D) HEK293T cells were either not transfected or transiently transfected with wt *ATF4* reporter, treated with DMSO (control) for 3 h and total RNA was isolated. qPCR product levels of the *ATF4* mRNA obtained from Amplicon 2 (left panel) or Amplicon 3 (right panel) were normalized to the internal RNA isolation control (SPIKE) and compared between non-transfected and transiently transfected cell with non-transfection values set to 1 (*p<0.01). Results are representative of three independent experiments.
(E) Disome-seq coverage along the *ATF4* locus analyzed by RiboCrypt tool (https://ribocrypt.org). Mapped reads were reduced to the 5‘ end of the reads to increase resolution and facilitate identification of the individual peaks. Top track: eIF5A depletion; bottom track: wild type. “Disome peaks” indicates the 5’ ends of disomes; “CUG” and “SL3” indicate their respective positions; “Amplicons” depict the regions subjected to RT-qPCR in Figure 6 and panels A – C.

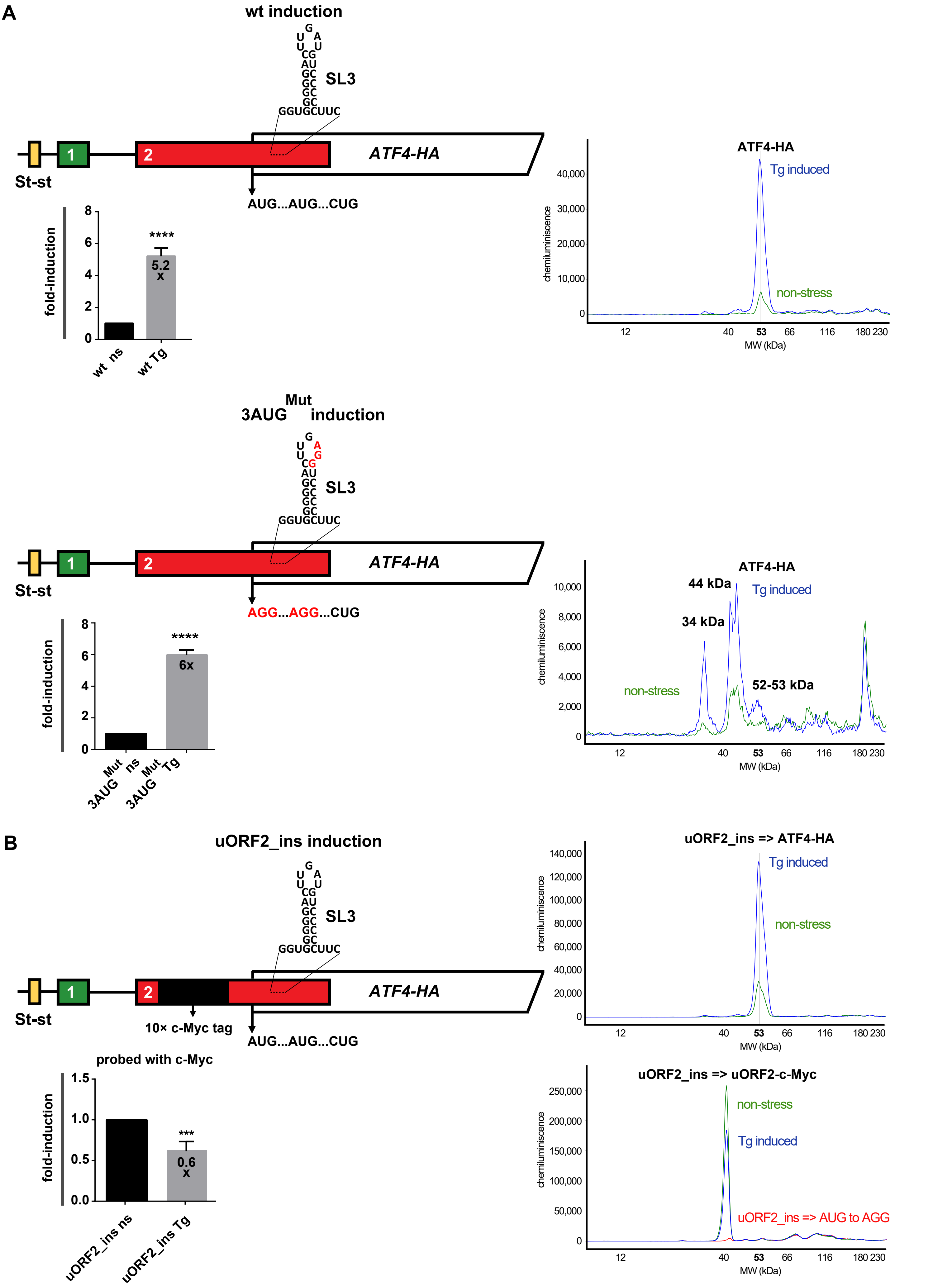
A
wt induction
SL3
GGUGCUUC
St-st
1
2
ATF4-HA
AUG...AUG...CUG
fold-induction
****
5.2 x
wt ns
wt Tg
ATF4-HA
Tg induced
non-stress
chemiluminiscence
40,000
30,000
20,000
10,000
0
12
40
53
66
116
180 230
MW (kDa)
Mut 3AUG induction
SL3
GGUGCUUC
St-st
1
2
ATF4-HA
AGG...AGG...CUG
fold-induction
****
6x
Mut 3AUG ns
Mut 3AUG Tg
ATF4-HA
44 kDa
Tg induced
34 kDa
non-stress
52-53 kDa
chemiluminiscence
10,000
8,000
6,000
4,000
2,000
0
MW (kDa)
B
uORF2_ins induction
SL3
GGUGCUUC
St-st
1
2
ATF4-HA
10× c-Myc tag
AUG...AUG...CUG
probed with c-Myc
fold-induction
1.5
1.0
0.5
0.0
***
0.6 x
uORF2_ins ns
uORF2_ins Tg
uORF2_ins => ATF4-HA
Tg induced
non-stress
chemiluminiscence
140,000
120,000
100,000
80,000
60,000
40,000
20,000
0
MW (kDa)
uORF2_ins => uORF2-c-Myc
non-stress
Tg induced
uORF2_ins => AUG to AGG
250,000
200,000
150,000
100,000
50,000
0
MW (kDa)

Figure S7*. The *ATF4* mRNA expression is not subject to frameshifting; uORF2 is translated even under stress (related to text in Methods Details)**.**

(A) Same as Figure 1E for better comparison except that the $3AUG^{Mut}$ construct (bottom panel; depicted at its the top) was also subjected to JESS analyses. The electropherograms of the wt (top panel) and the $3AUG^{Mut}$ mutant construct (bottom panel) under Tg stress (in blue) compared to non-stress conditions (in green) probed with anti-HA antibodies are shown (n≥3). For details, see the main text.

(B) Same as in Figure S3A except that the 10x c-Myc tag insertion in-frame with uORF2, depicted at the top of the panel, was subjected to JESS analyses (n≥3). The level of uORF2 induction under stress determined by anti-c-Myc antibodies is plotted. The electropherograms of the construct bearing the 10x c-Myc tag insertion in-frame with uORF2 under Tg stress (in blue) compared to non-stress conditions (in green) probed with the anti-HA (top panel) and anti-c-Myc (bottom panel) antibodies are shown. The electropherogram of the control construct bearing the 10x c-Myc tag insertion in-frame with uORF2, the AUG of which was mutated to AGG, is shown in red. For details, see the main text.

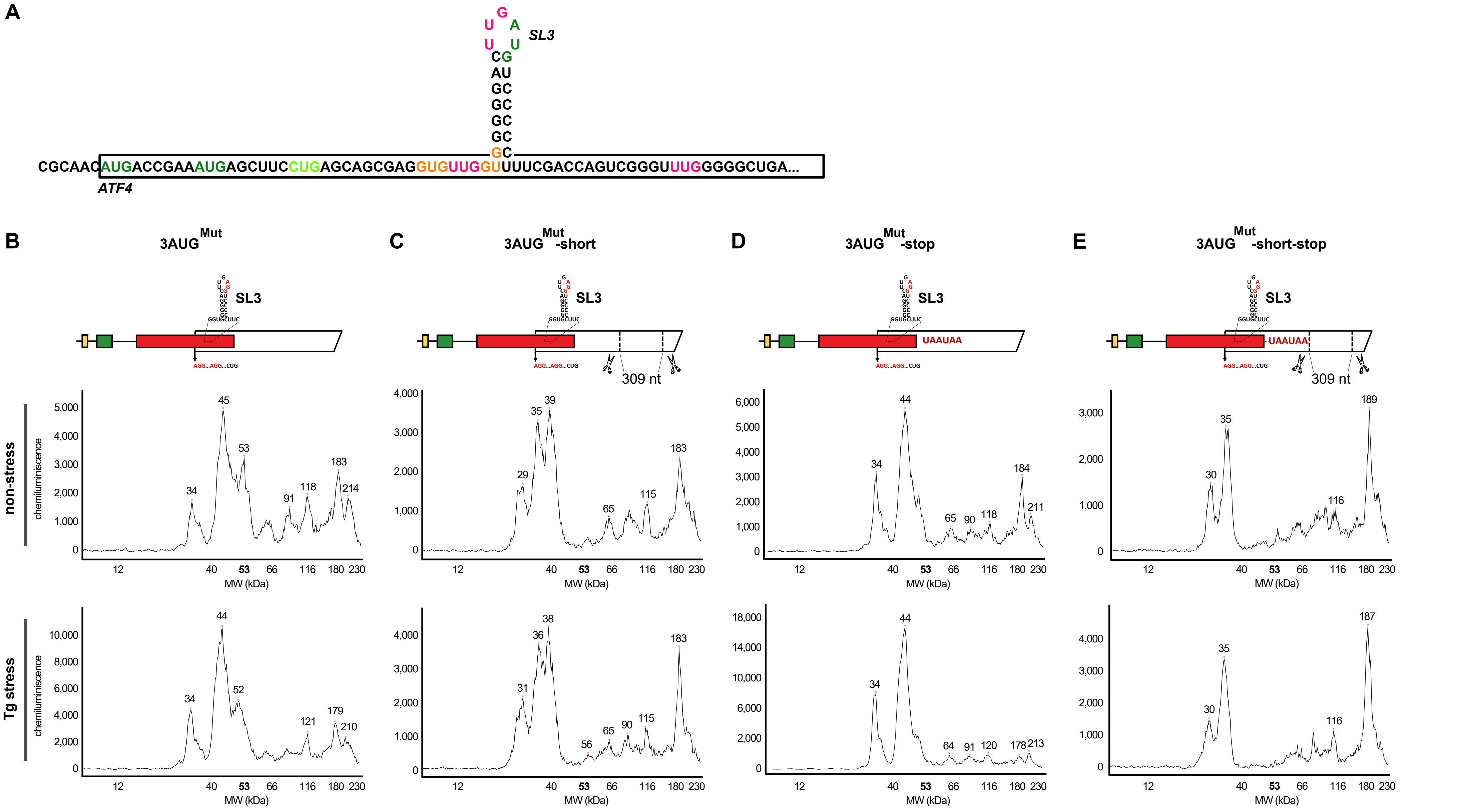
A
SL3
CGCAACAUGACCGAAAUGAGCUUCCUGAGCAGCGAGGUGUUGGUUUUCGACCAGUCGGGUUUGGGGGCUGA...
ATF4
B
3AUG Mut
C
3AUG Mut -short
D
3AUG Mut -stop
E
3AUG Mut -short-stop
SL3
AGG...AGG...CUG
309 nt
UAAUAA
non-stress
Tg stress
chemiluminiscence
MW (kDa)

Figure S8. The *ATF4* mRNA expression is not subject to frameshifting; the canonical AUG1 translation start site of ATF4 is substantial leaky scanned (related to text in Methods Details)**.**

(A) Schematic representation of the *ATF4* sequence showing the annotated AUG translation start site (TSS) of the ATF4 full-length protein, two alternative canonical TSSs, near-cognate CUG and five other near-cognate codons in uORF2/*ATF4* overlap.

(B – E) The electropherograms of the full length (B) or C-terminally shortened (C) *ATF4* constructs bearing $3AUG^{Mut}$ without (B – C) or with 2 consecutive UAA stops inserted immediately downstream of the uORF2/*ATF4* overlap (D – E), depicted at the top of the corresponding panels, are shown.

Smirnova Fig. S9

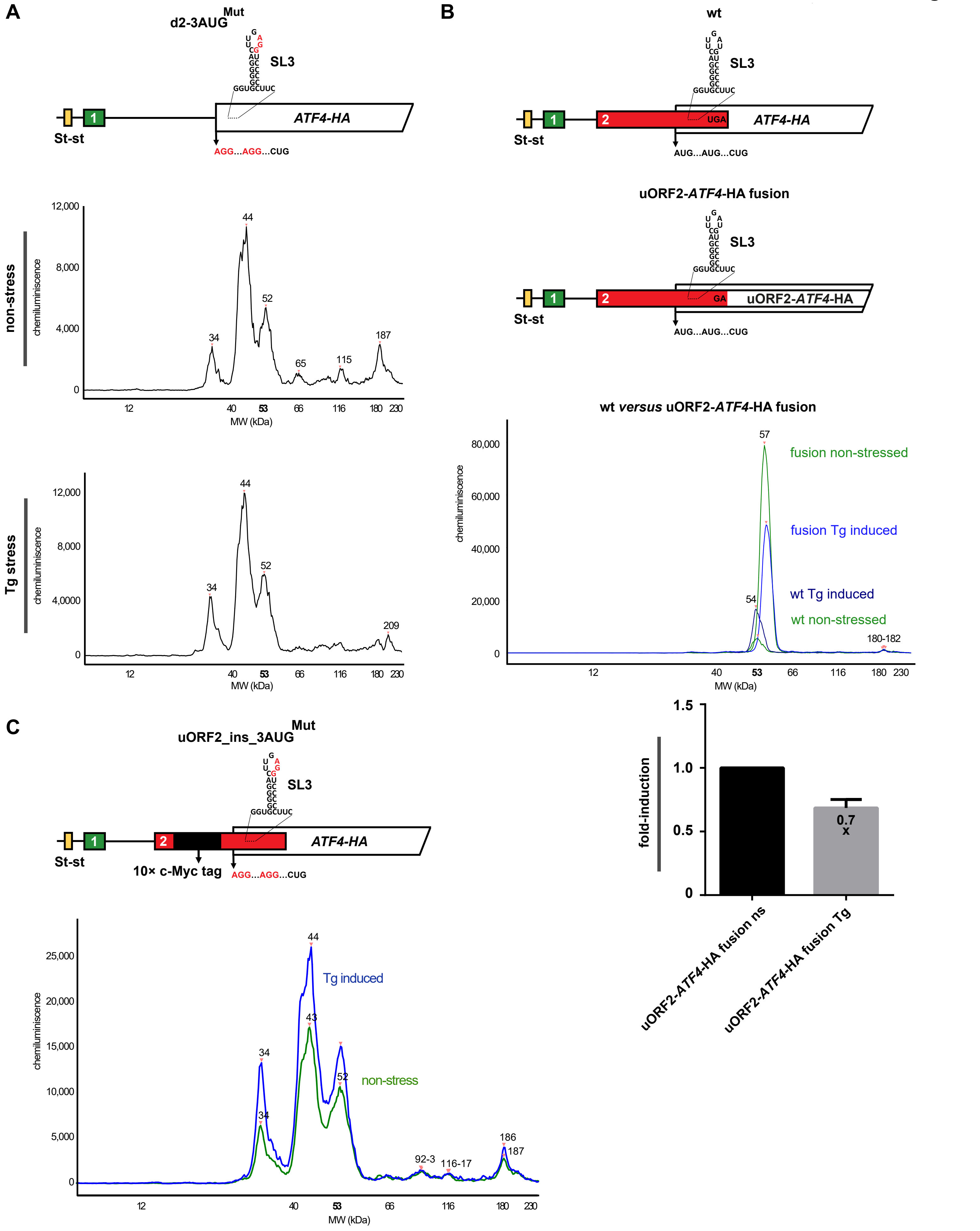

Figure S9. Supporting mutational analysis of the *ATF4* mRNA leader (related to Figure 1 and text in Methods Details)**.**

(A) Combining $3AUG^{Mut}$ with the AUG to AGG mutation of uORF2 shows no impact on the size and distribution of the ATF4 protein variants expressed with $3AUG^{Mut}$ alone. The electropherogram of the $3AUG^{Mut}$ mutation combined with the AUG to AGG mutation of uORF2 in the same *ATF4* construct, depicted at the top, is shown.

(B) The artificially created uORF2-*ATF4*-HA fusion product further confirms sustained uORF2 expression even under stress conditions. Schematics at the top depict wt and engineered uORF2-*ATF4*-HA fusion constructs with the electropherograms indicating their expression under non-stress (shades of green) *versus* stress (shades of blue) conditions. Fold-induction values of the fusion construct were plotted (bottom panel) further confirming uORF2 expression even under stress conditions (n=2).

(C) Further evidence that the *ATF4* mRNA expression is not subject to frameshifting and that uORF2 is expressed even under stress conditions. The schematic at the top depicts the *ATF4*-HA mutant construct where $3AUG^{Mut}$ was combined with 10x c-Myc tag insertion in-frame with uORF2 with the electropherograms indicating their expression under non-stress (green) *versus* stress (blue) conditions.

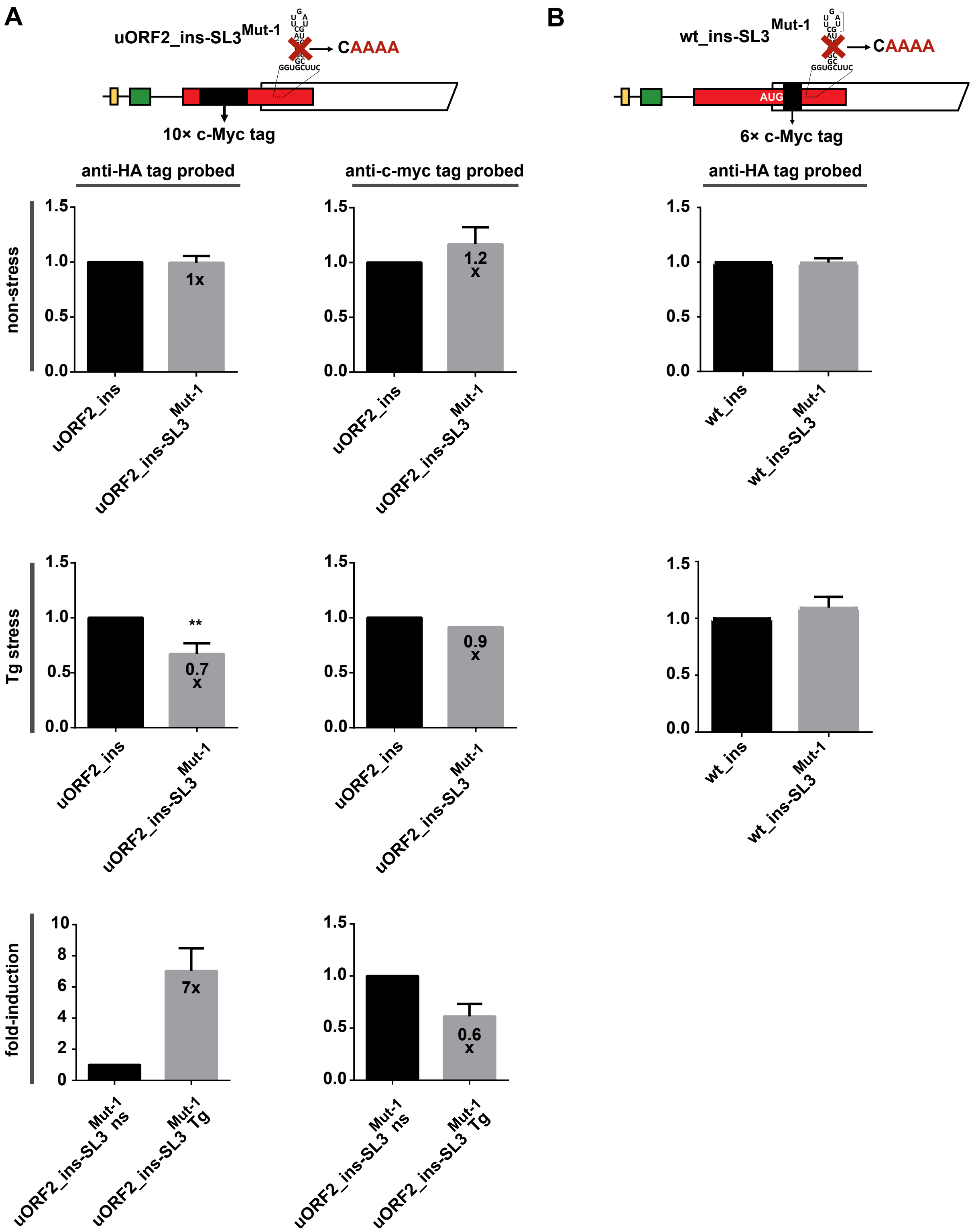
A
uORF2_ins-SL3Mut-1
CAAAA
GGUGCUUC
10× c-Myc tag
anti-HA tag probed
anti-c-myc tag probed
non-stress
Tg stress
fold-induction
1x
1.2 x
**
0.7 x
0.9 x
7x
0.6 x
uORF2_ins
uORF2_ins-SL3 Mut-1
uORF2_ins-SL3 Mut-1 ns
uORF2_ins-SL3 Mut-1 Tg
B
wt_ins-SL3Mut-1
AUG
6× c-Myc tag
wt_ins
wt_ins-SL3 Mut-1

Figure S10. Precise placement of SL3 inside of uORF2 with an exactly defined length expands the existing delayed REI model to a more complex model that includes ribosome queuing (related to Figure 4)**.**

(A) The 10x c-Myc tag insertion in-frame with uORF2 was combined with the $SL3^{Mut-1}$ mutation, as depicted at the top, and subjected to JESS analyses as described in Figure 1E. Probing with both anti-HA (left plots, n≥2) and anti-c-Myc (right plots) antibodies are shown.

(B) The 6x c-Myc tag insertion in-frame with ATF4 was combined with the $SL3^{Mut-1}$ mutation, as depicted at the top, and subjected to JESS analyses (n=3) as described in Figure 1E.

SUPPLEMENTAL TABLES

Table S1. Related to **Figure 1E** and **1F**; relative wt ATF4-HA protein expression under 3 hours of thapsigargin (Tg) or 4 hours of tunicamycin stress compared to wt ATF4-HA under non-stress (ns) set to 1.

	**wt** Tg **(N, p-value)**
**fold-induction**	5.21 ± 0.51 (17, < 0.0001 ****)

	**wt** tunicamycin **(N, p-value)**
**fold-induction**	2.91 ± 0.50 (3, 0.0027 **)

Table S2. Related to **Figure 1E** and **1F** and **Table S1**; relative wt ATF4-HA protein expression under 3 hours of thapsigargin (Tg) or 4 hours of tunicamycin stress compared to wt ATF4-HA under non-stress (ns) set to 1 – individual values.

	**wt** Tg
**fold-induction**	5.7864
	5.0408
	5.5577
	4.7770
	5.6732
	4.9854
	4.5372
	4.3654
	5.3362
	4.5333
	6.1444
	4.7124
	5.4342
	5.8214
	5.1763
	5.1763
	5.5115

	**wt** tunicamycin
**fold-induction**	3.1259
	2.3398
	3.2610

Table S3. Related to **Figure S3**; relative ATF4-HA protein expression with wt set to 1. For wt ATF4-HA tag fold-induction see Table S1.

	**d-all (N, p-value)**
**non-stress**	22.68 ± 2.45 (5, <0.0001 ****)
**Tg stress**	3.31 ± 0.49 (3, 0.0012**)
**fold-induction**	1.03 ± 0.04 (3, 0.3357 ns)
	**St-st-only (N, p-value)**
**non-stress**	20.56 ± 3.43 (3, 0.0006 ***)
**Tg stress**	3.05 ± 0.41 (3, 0.0010 ***)
**fold-induction**	0.93 ± 0.11 (3, 0.3064 ns)
	**u1-only (N, p-value)**
**non-stress**	12.22 ± 2.17 (3, 0.0009 ***)
**Tg stress**	2.67 ± 0.29 (3, 0.0006 ***)
**fold-induction**	0.95 ± 0.04 (3, 0.1438 ns)
	**u2-only (N, p-value)**
**non-stress**	0.19 ± 0.03 (4, <0.0001 ****)
**Tg stress**	0.23 ± 0.04 (3, <0.0001 ****)
**fold-induction**	3.24 ± 0.34 (3, 0.0003 ***)
	**dSt-st (N, p-value)**
**non-stress**	0.96 ± 0.11 (6, 0.3801 ns)
**Tg stress**	0.95 ± 0.05 (3, 0.1221 ns)
**fold-induction**	5.77 ± 0.33 (3, <0.0001 ****)
	**d1 (N, p-value)**
**non-stress**	0.29 ± 0.01 (3, <0.0001 ****)
**Tg stress**	0.33 ± 0.06 (3, <0.0001 ****)
**fold-induction**	5.81 ± 0.41 (3, <0.0001 ****)
	**d2 (N, p-value)**
**non-stress**	12.08 ± 2.15 (7, <0.0001 ****)
**Tg stress**	2.43 ± 0.16 (6, <0.0001 ****)
**fold-induction**	0.98 ± 0.06 (4, 0.5065 ns)

Table S4. Related to **Figure S3** and **Table S3**; relative ATF4-HA protein expression with wt set to 1 – individual values. For wt ATF4-HA tag fold-induction see Table S2.

	**d-all**	**St-st-only**	**uORF1-only**	**uORF2-only**
**non-stress**	20.2108<br>20.1493<br>24.9716<br>25.1497<br>22.9260	19.2065<br>24.4600<br>18.0205	11.7912<br>14.5744<br>10.2913	0.2182<br>0.1407<br>0.2058<br>0.1765
**Tg stress**	2.8184<br>3.3113<br>3.7961	2.5967<br>3.1636<br>3.3866	2.3353<br>2.8305<br>2.8535	0.2745<br>0.2188<br>0.1998
**fold-induction**	0.9906<br>1.0773<br>1.0164	0.8076<br>0.9861<br>0.9930	0.9920<br>0.9045<br>0.9633	2.8828<br>3.2869<br>3.5573
	**dSt-st**	**d1**	**d2**	
**non-stress**	0.9732<br>0.8915<br>0.8889<br>0.8518<br>1.0173<br>1.1380	0.2875<br>0.2954<br>0.2919	14.0385<br>15.4196<br>8.8531<br>11.3319<br>10.7310<br>12.0588<br>12.1492	
**Tg stress**	0.9235<br>1.0011<br>0.9185	0.3767<br>0.3440<br>0.2564	2.5079<br>2.4676<br>2.5781<br>2.2401<br>2.5556<br>2.2038	
**fold-induction**	6.1441<br>5.5343<br>5.6200	6.2766<br>5.6519<br>5.5149	0.9584<br>0.9709<br>1.0598<br>0.9330	

Table S5. Related to **Figure 3B**; relative ATF4-HA protein expression with wt (or parental d2) constructs set to 1. For wt ATF4-HA tag fold-induction see Table S1, for d2 fold-induction – Table S3.

	**wt-SL3^{Mut-1} (N, p-value)**	**wt- SL3^{Mut-2} (N, p-value)**
**non-stress**	1.31 ± 0.13 (5, 0.0007 ***)	1.72 ± 0.16 (4, 0.0001 ***)
**Tg stress**	1.53 ± 0.28 (6, 0.0022 **)	1.84 ± 0.22 (3, 0.0027 **)
**fold-induction**	5.69 ± 0.59 (6, < 0.0001 ****)	5.87 ± 0.18 (3, < 0.0001 ****)
	**d2-SL3^{Mut-1} (N, p-value)**	
**non-stress**	1.69 ± 0.33 (6, 0.0022 **)	
**Tg stress**	1.40 ± 0.21 (5, 0.0027 **)	
**fold-induction**	0.99 ± 0.12 (7, 0.8315 ns)	

Table S6. Related to **Figure 3B** and **Table S5**; relative ATF4-HA protein expression with wt (or parental d2) construct set to 1 – individual values. For wt ATF4-HA tag fold-induction see Table S2, for d2 fold-induction – Table S4.

	**wt-SL3^{Mut-1}**	**d2-SL3^{Mut-1}**	**wt-SL3^{Mut-2}**
**non-stress**	1.3549<br>1.3636<br>1.1961<br>1.1578<br>1.4705	1.3556<br>1.9470<br>1.3943<br>1.4266<br>2.0385<br>1.9983	1.5097<br>1.6794<br>1.7911<br>1.8890
**Tg stress**	1.4009<br>1.5382<br>1.2837<br>1.4387<br>1.4448<br>2.0767	1.1336<br>1.5362<br>1.6662<br>1.3437<br>1.3058	1.9355<br>1.9997<br>1.5906
**fold-induction**	4.7580<br>5.2583<br>6.2353<br>5.6282<br>6.2162<br>6.0593	0.8509<br>0.8849<br>1.0014<br>0.9475<br>0.9471<br>1.1995<br>1.0983	5.6622<br>5.9618<br>5.9843

Table S7. Related to **Figure 4B**; relative ATF4-HA protein expression with wt set to 1. For wt ATF4-HA tag fold-induction see Table S1; for average values of wt-SL3^{Mut-1} construct see Table S5.

	**wt-CUG^{Mut-1} (N, p-value)**	**wt-SL3^{Mut-1}-CUG^{Mut} (N, p-value)**
**non-stress**	0.78 ± 0.07 (3, 0.0072 **)	1.04 ± 0.07 (3, 0.4482 ns)
**Tg stress**	0.81 ± 0.06 (4, 0.0009 ***)	1.13 ± 0.11 (3, 0.1065 ns)
**fold-induction**	5.56 ± 0.59 (3, 0.0002 ***)	5.40 ± 0.33 (3, <0.0001****)

Table S8. Related to **Figure 4B** and **Table S7**; relative ATF4-HA protein expression with wt set to 1 – individual values. For wt ATF4-HA tag fold-induction see Table S2; for individual values of wt-SL3^{Mut-1} construct see Table S6.

	**WT-CUG^{Mut} 8h**	**WT-SL3^{Mut-1}-CUG^{Mut} 8h**
**non-stress**	0.8477<br>0.8005<br>0.7022	0.9834<br>1.0037<br>1.1208
**Tg stress**	0.8775<br>0.7303<br>0.8225<br>0.8214	1.0397<br>1.0960<br>1.2442
**fold-induction**	6.1537<br>5.5542<br>4.9730	5.7668<br>5.2947<br>5.1425

Table S9. Related to **Figure 5**; relative ATF4-HA protein expression with wt (or parental, i.e., wt-SL3^{Mut} or d2, or d2-SL3^{Mut}) constructs set to 1. For wt ATF4-HA tag fold-induction see Table S1; for d2 – Table S3; for wt-SL3^{Mut} and d2-SL3^{Mut} constructs – Table S5.

	**wt_ins (N, p-value)**	**d2_ins (N, p-value)**
**non-stress**	1.39 ± 0.14 (5, 0.0002 ***)	1.81 ± 0.28 (4, 0.0011 **)
**Tg stress**	1.34 ± 0.22 (4, 0.0219 *)	1.43 ± 0.31 (6, 0.0067 **)

	**wt_ins_SL3^{Mut-1} (N, p-value)**	**d2_ins_SL3^{Mut-1} (N, p-value)**
**non-stress**	0.99 ± 0.10 (4, 0.7780 ns)	1.59 ± 0.30 (6, 0.0007 ***)
**Tg stress**	1.02 ± 0.11 (4, 0.7349 ns)	1.26 ± 0.32 (8, 0.0002 ***)

Table S10. Related to **Figure 5** and **Table S9**; relative ATF4-HA protein expression with wt (or parental, i.e., wt-SL3^{Mut} or d2, or d2-SL3^{Mut}) constructs set to 1 – individual values. For wt ATF4-HA tag fold-induction see Table S2; for d2 – Table S4; for wt-SL3^{Mut} or d2-SL3^{Mut} constructs see Table S6.

	**WT_ins**	**WT_ins_SL3^{Mut-1}**	**d2_ins**	**d2_ins_SL3^{Mut-1}**
**non-stress**	1.4490<br>1.2790<br>1.2500<br>1.5890<br>1.3870	0.9865<br>1.0112<br>0.8528<br>1.0911	1.6911<br>1.7999<br>1.5545<br>2.1982	1.7029<br>1.7096<br>1.0144<br>1.5409<br>1.7194<br>1.8803
**Tg stress**	1.6742<br>1.1828<br>1.2674<br>1.2488	1.0010<br>1.0360<br>1.1540<br>0.8870	1.2788<br>1.6109<br>1.2160<br>1.0305<br>1.8753<br>1.5836	1.0850<br>1.0152<br>1.4790<br>1.3575<br>1.0399<br>1.0243<br>1.1518<br>1.9221

Table S11. Related to **Figure S7**; relative 3AUG^{Mut} (or uORF2_ins) protein expression under 3 hours of thapsigargin (Tg) stress compared to 3AUG^{Mut} (or uORF2_ins, respectively) under non-stress (ns) set to 1. For wt ATF4-HA tag fold-induction see Table S1.

	**3AUG^{Mut} (N, p-value)**	**uORF2_ins (N, p-value)**
**fold-induction**	5.98 ± 0.31 (3, <0.0001 ****)	0.62 ± 0.12 (4, 0.0006 ***)

Table S12. Related to **Figure S7** and **Table S11**; relative 3AUG^{Mut} (or uORF2_ins) protein expression under 3 hours of thapsigargin (Tg) stress compared to 3AUG^{Mut} (or uORF2_ins, respectively) under non-stress (ns) set to 1 – individual values. For wt ATF4-HA tag fold-induction see Table S2.

	**3AUG^{Mut}**	**uORF2_ins**
**fold-induction**	5.7269	0.7550
	6.3253	0.6555
	5.8946	0.4766
		0.6090

Table S13. Related to **Figure S9B**; relative uORF2-HA fusion protein expression under 3 hours of thapsigargin (Tg) stress compared to uORF2-HA under non-stress (ns) set to 1.

	**uORF2-HA Tg (N)**
**fold-induction**	0.69 ± 0.07 (2)

Table S14. Related to **Figure S9B** and **Table S13**; relative uORF2-HA fusion protein expression under 3 hours of thapsigargin (Tg) stress compared to uORF2-HA under non-stress (ns) set to 1 – individual values.

	**uORF2-HA Tg**
**fold-induction**	0.7324
	0.6388

Table S15. Related to **Figure S10A**; relative ATF4-HA protein expression with uORF2_ins set to 1. For uORF2_ins fold-induction see Table S11.

	**uORF2_ins-SL3^{Mut-1} (N, p-value)**	
	**anti-HA tag probed**	**anti-c-myc tag probed**
**non-stress**	1.00 ± 0.06 (2)	1.17 ± 0.16 (2)
**Tg stress**	0.67 ± 0.10 (3, 0.0043 **)	0.91 (1)
**fold-induction**	7.04 ± 1.45 (2)	0.61 ± 0.12 (2)

Table S16. Related to **Figure S10A** and **Table S15**; relative ATF4-HA protein expression with uORF2_ins set to 1 – individual values. For uORF2_ins fold-induction see Table S11.

	**uORF2_ins-SL3^{Mut-1}**	
	**anti-HA tag probed**	**anti-c-myc tag probed**
**non-stress**	1.0382<br>0.9510	1.0564<br>1.2776
**Tg stress**	0.5718<br>0.6703<br>0.7681	0.9147
**fold-induction**	6.0108<br>8.0632	0.6978<br>0.5295

Table S17. Related to **Figure S10B**; relative ATF4-HA protein expression with wt_ins set to 1.

	**wt_ ins-SL3^{Mut-1} (N, p-value)**
**non-stress**	1.00 ± 0.04 (3, 0.8895 ns)
**Tg stress**	1.09 ± 0.09 (3, 0.1568 ns)

Table S18. Related to **Figure S10B** and **Table S17**; relative ATF4-HA protein expression with wt_ins set to 1 – individual values.

	**wt_ ins-SL3^{Mut-1}**
**non-stress**	0.9781<br>0.9699<br>1.0419
**Tg stress**	0.9963<br>1.1847<br>1.1039

Table S19. Related to **Figure 7B** (left and middle panels); relative ATF4-HA protein expression with wt set to 1. For wt ATF4-HA tag fold-induction see Table S1.

	**wt-A*235G (N, p-value)**	**wt-A*326G (N, p-value)**
**non-stress**	1.34 ± 0.06 (4, < 0.0001 ****)	0.78 ± 0.09 (4, 0.0032 **)
**Tg stress**	1.10 ± 0.15 (3, 0.2960 ns)	0.83 ± 0.16 (4, 0.0681 ns)
**fold-induction**	6.22 ± 0.57 (4, < 0.0001 ****)	4.83 ± 0.55 (4, < 0.0001 ****)

Table S20. Related to **Figure 7B** (left and middle panels) and **Table S17**; relative ATF4-HA protein expression with wt set to 1 – individual values. For wt ATF4-HA tag fold-induction see Table S2.

	**wt-A*235G**	**wt-A*326G**
**non-stress**	1.3842<br>1.4059<br>1.2710<br>1.3182	0.6764<br>0.7224<br>0.8623<br>0.8529
**Tg stress**	0.9654<br>1.2600<br>1.0831	0.8514<br>0.6066<br>0.8833<br>0.9687
**fold-induction**	6.4411<br>5.7568<br>6.9359<br>5.7596	5.5512<br>4.3221<br>4.4940<br>4.9439

Table S21. Related to **Figure 7C**; relative levels of amplification efficiencies of *ATF4* transcript regions containing either A_{235} or A_{326} in mock treated *ATF4* mRNA with Tg treated set to 1.

**HEK293T**	**A_{235} (N, p-value)**	**A_{326} (N, p-value)**
**5 min**	0.5025 ± 0.0990 (4, 0.0032 **)	0.8725 ± 0.0192 (4, 0.0014 **)
**15 min**	0.5275 ± 0.0629 (4, 0.001 ***)	0.92 ± 0.0604 (4, 0.1056 ns)
**60 min**	0.95 ± 0.2152 (4, 0.7144 ns)	0.8475 ± 0.0675 (4, > 0.0298 *)
**HeLa**	**A_{235} (N, p-value)**	**A_{326} (N, p-value)**
**5 min**	0.4325 ± 0.1229 (4, 0.0041 **)	0.89 ± 0.2410 (4, 0.4870 ns)
**15 min**	0.3013 ± 0.1298 (4, 0.0026 **)	0.85 ± 0.2178 (4, 0.3187 ns)
**60 min**	0.5025 ± 0.3338 (4,>0.0817 ns)	0.995 ± 0.1265 (4, > 0.9498 ns)

Table S22. Related to **Figure 7B** (right panel); relative ATF4-HA protein expression with wt set to 1.

	**wt-SL3^{Mut-1}-A*326G (N, p-value)**
**non-stress**	0.86 ± 0.04 (3, 0.0044 **)
**Tg stress**	1.59 ± 0.16 (3, 0.0032 **)
**fold-induction**	6.48 ± 0.34 (3, < 0.0001 ****)

Table S23. Related to **Figure 7B** (right panel) and **Table S22**; relative ATF4-HA protein expression with wt set to 1 – individual values.

	**wt-SL3^{Mut-1}-A*326G**
**non-stress**	0.8557<br>0.8275<br>0.9078
**Tg stress**	1.7344<br>1.4163<br>1.6309
**fold-induction**	6.8769<br>6.2719<br>6.3050

SUPPLEMENTARY REFERENCES

1. Madeira, F., Pearce, M., Tivey, A.R.N., Basutkar, P., Lee, J., Edbali, O., Madhusoodanan, N., Kolesnikov, A., and Lopez, R. (2022). Search and sequence analysis tools services from EMBL-EBI in 2022. Nucleic Acids Res *50*, W276-W279. 10.1093/nar/gkac240.
2. Rendleman, J., Cheng, Z., Maity, S., Kastelic, N., Munschauer, M., Allgoewer, K., Teo, G., Zhang, Y.B.M., Lei, A., Parker, B., et al. (2018). New insights into the cellular temporal response to proteostatic stress. Elife *7*. 10.7554/eLife.39054.
3. Kolde, R., and Kolde, M. (2015). R Package ‚pheatmap'. R package 1. web tool *790*.
4. Lauria, F., Tebaldi, T., Bernabo, P., Groen, E.J.N., Gillingwater, T.H., and Viero, G. (2018). riboWaltz: Optimization of ribosome P-site positioning in ribosome profiling data. PLoS Comput Biol *14*, e1006169. 10.1371/journal.pcbi.1006169.
5. Ichihara, K., Matsumoto, A., Nishida, H., Kito, Y., Shimizu, H., Shichino, Y., Iwasaki, S., Imami, K., Ishihama, Y., and Nakayama, K.I. (2021). Combinatorial analysis of translation dynamics reveals eIF2 dependence of translation initiation at near-cognate codons. Nucleic Acids Res *49*, 7298-7317. 10.1093/nar/gkab549.
6. Andreev, D.E., O'Connor, P.B., Fahey, C., Kenny, E.M., Terenin, I.M., Dmitriev, S.E., Cormican, P., Morris, D.W., Shatsky, I.N., and Baranov, P.V. (2015). Translation of 5' leaders is pervasive in genes resistant to eIF2 repression. Elife *4*, e03971. 10.7554/eLife.03971.
